# Computed Tomography to Exclude Cardiac Thrombus in Atrial Fibrillation—An 11-Year Experience from an Academic Emergency Department

**DOI:** 10.3390/diagnostics14070699

**Published:** 2024-03-27

**Authors:** Sophie Gupta, Martin Lutnik, Filippo Cacioppo, Teresa Lindmayr, Nikola Schuetz, Elvis Tumnitz, Lena Friedl, Magdalena Boegl, Sebastian Schnaubelt, Hans Domanovits, Alexander Spiel, Daniel Toth, Raoul Varga, Marcus Raudner, Harald Herkner, Michael Schwameis, Jan Niederdoeckl

**Affiliations:** 1 Department of Emergency Medicine, Medical University of Vienna, 1090 Vienna, Austria; sophie.gupta@meduniwien.ac.at (S.G.); filippo.cacioppo@meduniwien.ac.at (F.C.); teresa.lindmayr@meduniwien.ac.at (T.L.); nikola.schuetz@meduniwien.ac.at (N.S.); elvis.tumnitz@meduniwien.ac.at (E.T.); lena.friedl@inode.at (L.F.); magdalena.boegl@meduniwien.ac.at (M.B.); sebastian.schnaubelt@meduniwien.ac.at (S.S.); hans.domanovits@meduniwien.ac.at (H.D.); harald.herkner@meduniwien.ac.at (H.H.); jan.niederdoeckl@meduniwien.ac.at (J.N.); 2 Department of Clinical Pharmacology, Medical University of Vienna, 1090 Vienna, Austria; martin.lutnik@meduniwien.ac.at; 3 Clinical Division of Gynaecologic Endocrinology and Reproductive Medicine, Medical University of Vienna, 1090 Vienna, Austria; 4 Department of Emergency Medicine, Clinic Ottakring, Vienna Healthcare Group, 1160 Vienna, Austria; alexander.spiel@gesundheitsverbund.at; 5 Department of Radiology, Medical University of Vienna, 1090 Vienna, Austria; daniel.toth@meduniwien.ac.at (D.T.); raoul.varga@meduniwien.ac.at (R.V.); marcus.raudner@meduniwien.ac.at (M.R.)

**Keywords:** cardioversion, left atrial appendage thrombus, atrial fibrillation, emergency medicine

## Abstract

Background: Computed tomography (CT) could be a suitable method for acute exclusion of left atrial appendage thrombus (LAAT) prior to cardioversion of atrial fibrillation (AF) and atrial flutter (AFL) at the emergency department. Our aim was to present our experiences with this modality in recent years. Methods: This registry-based observational study was performed at the Department of Emergency Medicine at the Medical University of Vienna, Austria. We studied all consecutive patients with AF and AFL who underwent CT between January 2012 and January 2023 to rule out LAAT before cardioversion to sinus rhythm was attempted. Follow-ups were conducted by telephone and electronic medical records. The main variables of interest were the rate of LAAT and ischemic stroke at follow-up. Results: A total of 234 patients (143 [61%] men; median age 68 years [IQR 57–76], median CHA_2_DS_2_-VASc 2 [IQR 1–4]) were analyzed. Follow-up was completed in 216 (92%) patients after a median of 506 (IQR 159–1391) days. LAAT was detected in eight patients (3%). A total of 163 patients (72%) in whom LAAT was excluded by CT were eventually successfully cardioverted to sinus rhythm. No adverse events occurred during their ED stay. All patients received anticoagulation according to the CHA_2_DS_2_-VASc risk stratification, and no patient had suffered an ischemic stroke at follow-up, resulting in an incidence risk of ischemic strokes of 0% (95% CI 0.0–1.2%). Conclusion: LAAT was rare in patients admitted to the ED with AF and AFL who underwent cardiac CT prior to attempted cardioversion. At follow-up, no patient had suffered an ischemic stroke. Prospective studies need to show whether this strategy is suitable for the acute treatment of symptomatic AF in the emergency setting.

## 1. Background

Atrial fibrillation (AF) is a common cardiac arrhythmia worldwide and a frequent cause of admission to the emergency department (ED) [1,2]. Patients admitted to the ED for AF are often highly symptomatic, and identifying those who are candidates for cardioversion (CV) to restore sinus rhythm is a critical aspect of acute AF management [3]. Delayed decision-making may impede timely access to cardioversion resources, leading to treatment delays and suboptimal outcomes.

However, CV carries an increased risk of thromboembolism and, hence, requires sufficient anticoagulation before and after the procedure and the exclusion of cardiac thrombi. [4,5] AF leads to a reduction in the contractility and function of the left atrium, as evidenced by decreased Doppler velocities and atrial enlargement. The remodeling changes associated with AF transform the left atrial appendage (LAA) into a stagnant pouch, increasing the risk of stasis and thrombus formation [6]. LAA thrombi (LAAT) may dislodge during the pharmacologic or electrical restoration of sinus rhythm, or reform after cardioversion as a result of left atrial stunning [7].

The current guidelines of the European Society of Cardiology (ESC) recommend transoesophageal echocardiography (TEE) to rule out LAA thrombi before CV [8]. However, TEE is resource-intensive, requires specialized staff, and may not be promptly available in an overcrowded ED. In rare cases, TEE can be associated with serious complications, such as oesophageal injury and sedation-related side effects [9,10]. In addition, some patients are not eligible for TEE due to relative contraindications, such as dysphagia, restricted neck mobility, oesophageal varices, or coagulopathy [11].

These limitations emphasize the need for alternative imaging modalities [12]. Computed tomography (CT) has proven to be a suitable method for detecting cardiac thrombi. There is evidence for the high diagnostic accuracy of CT in this context, especially for pulmonary vein isolation (PVI) and the exclusion of LAA thrombi [13]. However, there are limited data on the use of CT prior to cardioversion in the ED, and on long-term outcomes after this procedure [14]. 

With regard to the role of CT in pre-cardioversion assessment, it is important to consider the potential benefits and challenges associated with this imaging modality. CT offers several advantages over TEE, including non-invasiveness, a shorter procedure time, and a potentially lower risk of complications such as oesophageal injury. By providing detailed anatomical information about the heart and surrounding structures, CT can aid in the accurate identification of cardiac thrombi and support further treatment decisions, avoiding the need for another CT scan if PVI is planned [15].

In addition, integrating CT into the workflows of EDs for pre-cardioversion assessment could streamline care pathways and optimize resource utilization [16]. By reducing the reliance on TEE or other invasive procedures that may be logistically challenging or time-consuming in the acute care setting, CT imaging may expedite decision-making processes and facilitate timely interventions in patients with AF who require urgent treatment.

Despite these potential benefits, several issues must be considered when implementing CT for pre-cardioversion assessment in clinical practice. Technical expertise in the interpretation of cardiac imaging is essential to ensure an accurate diagnosis and appropriate management recommendations based on CT findings. Moreover, considerations regarding radiation exposure and the use of contrast agents should be carefully weighed against the diagnostic benefit of CT imaging in the pre-cardioversion assessment [15]. Strategies to minimize radiation dose while maintaining image quality should be implemented to minimize potential risks associated with repeated imaging examinations over time [17]. Monitoring renal function and optimizing contrast protocols are also important aspects of ensuring safety during CT examinations.

As CT has been used for thrombus detection prior to the cardioversion of AF and atrial flutter (AFL) in our ED for many years, our aim was to present our initial respective experiences with this modality.

## 2. Methods 

In this registry-based observational study, we investigated all episodes of AF and AFL treated between January 2012 and January 23 in the ED at the Medical University of Vienna, Austria, for which cardiac CT was performed prior to attempted cardioversion. The main outcomes were the rate of LAAT and ischemic stroke at follow-up. Furthermore, we analyzed the number of CT examinations to exclude a thrombus performed in recent years, particularly in relation to the total number of AF patients treated, the risk factors for the presence of LAAT on CT, and mortality at follow-up.

This study was approved by the local ethics committee and conducted in accordance with the Declaration of Helsinki. 

### 2.1. Study Centre and AF Registry 

The ED of the Medical University of Vienna, Austria, is an academic emergency facility for adult patients, consisting of an outpatient clinic and an intensive care unit. Approximately 90,000 patients are treated annually. The number of patients with AF and AFL treated each year ranges between 150 and 600. 

Since 2012, all patients with AF and AFL treated in our clinic have been registered in a database. Since the start of the registry, over 4000 episodes of AF and AFL have been documented. The standardized documentation contains information on demographics, medical history, home medication, clinical and laboratory parameters, and data relevant to AF (including CHA_2_DS_2_VASC risk stratification, type of arrhythmia, onset of arrhythmia, as well as information on the type of treatment, course of treatment, treatment success, discharge therapy, and follow-up examinations).

### 2.2. Follow-Up as Part of the Registry Database

Follow-up was primarily provided by telephone, and by consulting electronic medical records if communication with the patient was not possible. We used a standardized questionnaire in which the following information was requested: diagnosis of ischemic or hemorrhagic stroke, hospitalizations, recurrences of AF and AFL, and mortality. 

### 2.3. Study Population and CT Scan Protocol

In the present study, patients who were included in the AF registry and met the following five criteria were analyzed: (i) admission to the ED for AF or AFL, (ii) duration of symptoms could not be determined with certainty or symptoms had been present for more than 48 h, (iii) no adequate anticoagulation was present at the time of admission, (iv) cardioversion to restore sinus rhythm was chosen as the primary therapeutic strategy, and (v) an acute cardiac CT was performed to enable cardioversion.

The following cardiac CT protocol was used: All patients were examined with the same CT system (SOMOTOM Drive, Siemens Healthineers, Erlangen, Germany) in a supine position with arms held above the head. An ECG-triggered sequence of the whole heart was performed, followed by an additional delayed sequence of the atrium and atrial appendages. A total of 50 mL of an iodine-containing non-ionic monomeric contrast agent was used (Iomeron 400 mg/dL, Bracco, Milan, Italy).

If no thrombus was found on CT, cardioversion was performed according to clinical standards and current guidelines. Medical cardioversion (mCV) was performed with amiodarone, vernakalant, or ibutilide. Electrical cardioversion (eCV) was performed with biphasic defibrillators with increasing joule doses, starting with 100 joules to a maximum of 200 joules, with up to a maximum of three shocks. If eCV was initially unsuccessful, a second attempt was made after pharmacological pre-treatment. A sustained normal sinus rhythm during the ED stay was considered a therapeutic success. Transesophageal echocardiography or cardiac magnetic resonance imaging was not performed in any of the study patients. All patients received anticoagulation based on stroke risk stratification. Patients without existing anticoagulation received therapeutic anticoagulation according to individual patient characteristics. In patients with inadequate anticoagulation, the dose was adjusted accordingly. Which drug was used for anticoagulation was at the discretion of the treating physician.

### 2.4. Statistical Analysis

This study primarily contains a description of patients with AF and AFL who were treated in our ED and who underwent cardiac CT to diagnose a LAAT before attempting cardioversion, all subsequent analyses are exploratory. Continuous variables are presented as median with interquartile range (IQR). Categorical variables are presented as absolute numbers (n) and relative frequencies (%). We calculated the prevalence of LAAT and the 1-year incidence risk for ischemic stroke. We calculated 95% confidence intervals (95% CIs) for the proportions using Jeffrey’s interval. As a post-hoc sensitivity analysis, we restricted the analysis to patients who were included in the registry between the years 2021 and 2022. We used a Poisson model to calculate the overall mortality rate within 365 days together with a 95% CI. A logistic regression was performed to investigate possible risk factors for the presence of LAAT, where the effect is estimated as the odds ratio (OR) and the respective 95% CI. Since no ischemic strokes occurred during the follow-up period, inferential statistics using the outcome of ischemic stroke were not possible. Statistical significance was defined by two-sided *p*-values of <0.05. MS Excel 2021for Mac and STATA 17 for Mac were used for data analysis. 

## 3. Results

Since the start of the registry documentation, a total of 4283 episodes of AF and AFL have been recorded. Of these, 234 patients (5%) were included in the present analysis. The characteristics of the entire cohort of study patients are tabulated (Table 1) and presented for the subgroups of patients with and without LAAT, as well as for the overall study population.

### 3.1. LAAT Prevalence and AF Characteristics

Of the 234 patients enrolled in the study, eight were found to have LAAT on CT (prevalence 3.4%, 95% CI 1.6–6.3%). All of these patients received rate-controlling therapy. One patient converted to sinus rhythm spontaneously. Of the remaining 226 patients, 163 (72%) were eventually successfully converted to sustained sinus rhythm: 52 (43%) pharmacologically and 121 (74%) electrically; in 10 cases a combined approach of electrical and pharmacological cardioversion was used. Pharmacological cardioversion was performed with amiodarone, vernakalant or ibutilide. Electrical cardioversion (eCV) was performed with biphasic defibrillators with increasing joule doses, starting with 100 joules to a maximum of 200 joules, with up to a maximum of three shocks. In both groups, 38% (LAAT: n = 3; no LAAT: n = 87) were already on anticoagulation treatment at admission. The AF characteristics are presented in Table 2. 

In a sensitivity analysis, we restricted the inclusion time to the years 2021 and 2022. In this period, 358 patients presented with AF/AFL, among whom 192 (54%) had unclear onset or onset > 48 h, and 97 (51%) reported being on anticoagulation therapy, whereas the anticoagulation status was unclear in 10 (5%) patients. A total of 105 (45%) patients received a CT scan, where we found a prevalence of LAAT of 4.8% (95% CI 1.8–10.1).

### 3.2. Rate of Ischemic Stroke and Mortality

Follow-up was completed in 216 patients (92%). Within this period, we did not observe any stroke, resulting in an incidence risk of ischemic strokes of 0% (95% CI 0.0–1.2%). Among the 216 patients, a total of 14 patients (6%) died from causes other than stroke within 61,763 days of follow-up (median follow-up 365 days, IQR 158–365 days). This equals a mortality rate of 8.3 per 100 person–years (95% confidence interval 4.9–14.0). No deaths were observed in the group with LAAT. Table 3 shows the stroke and mortality rates as well as follow-up data.

### 3.3. Risk Factors for the Presence of LAAT

The comparisons between patients with and without LAAT are presented in Table 1. Among these comparisons, we found a statistically significant association between history of ischemic stroke and CT evidence of LAAT (OR 20.8 95% CI 1.59–271, *p* = 0.021). The odds ratio for the CHA_2_DS_2_-VASc score (0 or 1 versus > 1) as a risk factor for LAAT was 1.49 (95% CI 0.99–2.23, *p* = 0.053).

### 3.4. Temporal Trend of CT Scans Performed in the ED

**Figure 1:**
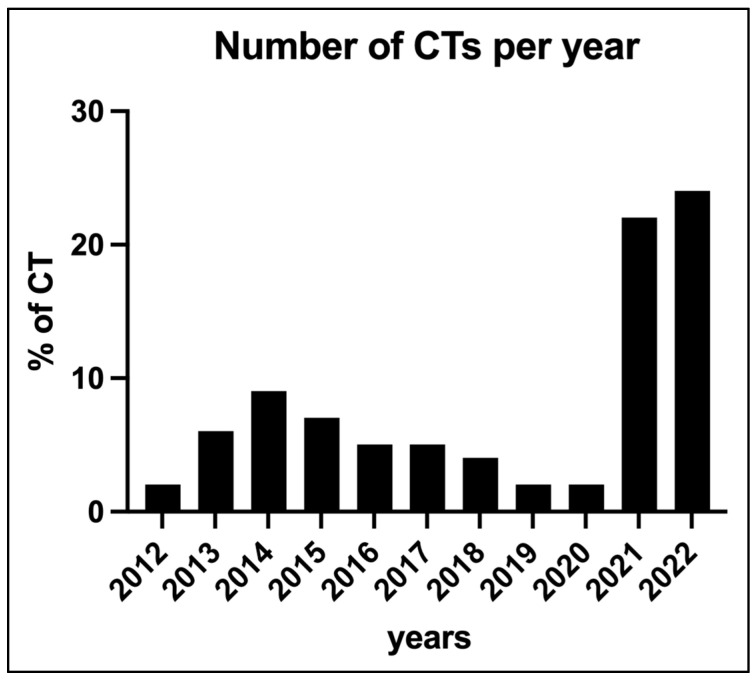
Annual CT examinations from 2011 to 2022 to rule out LAAT in patients with AF and AFL in the ED. *x*-axis: years. *y*-axis: percentage of all CT examinations to exclude LAAT since 2012.

Among the 234 CT scans performed since 2012, approximately half of the examinations (45%, n = 105) were performed since 2021 (Figure 1). In these years, a total of 358 episodes of AF were documented in the registry. In 105 (29%) of these episodes, a CT scan was performed with a specific protocol to diagnose LAAT.

## 4. Discussion

We present a collective of patients with AF and AFL who underwent CT in our ED before cardioversion was attempted. Given the limited applicability of respective guideline recommendations in the clinical practice of real-world emergency medicine, the use of this strategy has increased significantly in our center over the last 11 years. The evidence of CT for LAAT exclusion prior to acute cardioversion is still insufficient to recommend it as a general alternative. However, almost one-third of all patients (29%) with acute AF or AFL at our emergency department have been treated safely using our approach in the years since 2021. The LAAT rate observed was extremely low. None of the study patients had suffered acute complications or an ischaemic stroke in the period up until the follow-up procedure.

AF is the most common cardiac arrhythmia worldwide, associated with frequent recurrences, high levels of distress, and a significant impact on healthcare systems [1,2]. Especially in the setting of an emergency department, AF and AFL present a distinct challenge for treating physicians. Moreover, AF and AFL management in EDs has a big impact on outcomes, as respective patients have a higher hospitalization rate than patients admitted for any other reason than AF and AFL [18]. Insights from our ED reveal a higher mortality of AF and AFL patients in comparison to the general population [19]. The optimal acute treatment is still debated, although there is evidence that in certain patients rhythm restoration is associated with a more favorable outcome compared with a primary rate control strategy [20,21,22]. However, the iatrogenic restoration of sinus rhythm must not expose patients to an acute risk of thromboembolism [8]. AF increases the risk of stroke by a factor of three to five [23]. In addition, the risk of embolism increases after CV [5], with thrombi originating predominantly from the left atrium, and 90% of them originating from the left atrial appendage [23]. Therefore, focusing on those who are eligible for CV to consecutively restore sinus rhythm is a crucial part of AF management in EDs. 

If the restoration of sinus rhythm is chosen as the primary treatment strategy, an acute thrombus exclusion must be performed prior to cardioversion attempts in all patients without sufficient anticoagulation with a suspected symptom duration of >48 h or an unclear symptom onset [8]. Looking at our study population, these criteria include only 5% of all patients who were treated in our clinic in the last 11 years; however, this increased to almost a third in the past years. It is unclear whether this distribution is representative of other European EDs. However, in the context of the emergency care of patients with AF, there may be a significant need for an efficient, safe, and rapid thrombus exclusion procedure that can be pragmatically applied in daily practice. Patients with symptomatic AF who present to the ED often have a high level of distress and, in our experience, prefer the restoration of sinus rhythm to rate control in a joint decision-making process.

As mentioned above, patients undergoing CV are at an increased risk of stroke and thromboembolism, especially if they are not adequately anticoagulated. The exact duration of an AF episode prior to cardioversion can be difficult to determine, as many patients have asymptomatic AF and only seek medical attention when symptoms or complications arise. The mechanisms underlying the increased risk of peri-procedural thromboembolism include the presence of a pre-existing thrombus, changes in atrial mechanical function upon restoration of sinus rhythm, post-cardioversion atrial stunning, and a transient prothrombotic state. Moreover, there are data suggesting that a significant proportion of patients are inadequately anticoagulated [24,25]. Given the high number of patients in our study who were already undertaking oral anticoagulation (OAC) at the time of admission and still received thrombus exclusion, the importance of appropriate OAC dosing must be emphasized.

Due to its high diagnostic accuracy [26,27], TEE is recommended as the imaging procedure of choice to rule out LAAT in AF and AFL, both before pharmacological or electrical cardioversion and before pulmonary vein isolation (PVI) [8,28]. However, in order to be able to offer TEE at any time, the time and personnel resources of specially trained staff are required, which can be a relevant challenge, especially in EDs [16]. Furthermore, as a semi-invasive technique, TEE carries the risk of very rare but potentially serious complications, like oesophageal perforation, and has relative and absolute contraindications, including a history of dysphagia, restricted neck mobility, oesophageal varices, and coagulopathy [9,10,11,29].

CT for the exclusion of cardiac thrombi has been extensively studied in the context of PVI and has gained importance there. A recently published study of 260 patients showed that CT was not inferior to TEE in detecting atrial thrombi: 100% (n = 10) of thrombi were correctly identified by CT. Potential filling defects caused by blood stasis rather than thrombus could be accurately differentiated using a delayed imaging technique [30]. A meta-analysis published in 2013 that included 19 studies with 2955 patients mostly undergoing PVI found an overall accuracy of 94% for CT [15]. A recently published meta-analysis of 27 studies showed similar results and concluded that CT is a reliable alternative to TEE [31]. However, there are currently insufficient data on the diagnostic accuracy and safety of CT prior to the acute CV of patients with AF in EDs. To our knowledge, there are currently no large observational studies or randomized data on this topic. A previous small study analyzed a consecutive series of 52 patients with AF prior to CV, in which all cardiac thrombi (n = 7, 13%) were correctly identified [32]. In our study, LAAT was identified in eight patients (3%). The LAAT rate in our study was at the lower end of the prevalence distribution (2–25%) based on the results in the literature [15]. This is probably due to the characteristics of the patient populations in EDs. It should be kept in mind that the diagnostic accuracy of a test depends on the prevalence of a disease in a given setting.

Based on the available evidence, CT is likely to be a useful method for emergency physicians to safely perform CV. However, reliable data on the accuracy and safety of CT prior to cardioversion in the ED are needed to ensure optimal and safe patient care. 

### 4.1. Limitations

The present study is a largely descriptive presentation of a collective of patients with AF who were treated in our clinic and who underwent CT prior to attempted cardioversion. The main limitations are the retrospective study design, the small number of cases, the missing events, and the resulting analytical constraints. In addition, the time of the last follow-up was within a wide range, with the risk of stroke after cardioversion presumably being increased in the first four weeks in particular [33,34]. Our observations do not allow any conclusions to be drawn about the accuracy or safety of CT in the rhythm control of acute AF. In the context of the available evidence, our data suggest that CT may be a viable alternative to TEE in the emergency setting. However, it should be kept in mind that the rate of ischemic stroke in patients who are cardioverted without prior anticoagulation, but with subsequent anticoagulation, is low, and would not be captured by the limited study population and follow-up period [33].

In order to provide reliable evidence as to whether CT is a suitable alternative to TEE, large amounts of data are required to compare the risk–benefit ratio of both examinations. Additionally, CT is not risk-free, requires the intravenous administration of a contrast agent, and is associated with radiation exposure, the long-term effects of which are very difficult to determine, especially in individual cases. CT examinations necessitate the administration of approximately 60 to 120 mL of iodinated contrast agents, which pose a risk for contrast-induced nephropathy in patients with underlying kidney disease and risk factors such as diabetes mellitus, volume depletion, or advanced age. Similarly, in patients allergic to iodinated agents, CT may be relatively contraindicated, although premedication with H1 antihistamines and glucocorticoids can be considered. In addition, outsourcing thrombus diagnostics to the radiology department would save emergency physicians work, but increase the workload of radiologists.

It should be reiterated that the optimal therapeutic approach for patients with acute AF in the ED is currently unclear. Previous data suggest that there is no difference in outcome between rhythm and rate control [35,36]. In contrast, however, there are data that indicate that, in certain patients, rhythm restoration is associated with a more favorable outcome [20,21,22]. The decision on the therapeutic approach to AF is often made by the patient and physician on a case-by-case basis. Regardless of whether rate or rhythm control is chosen, the most efficient and safest approach for both parties should be pursued. 

### 4.2. Conclusions

LAAT was rare in patients admitted to the ED with AF and AFL who underwent cardiac CT prior to attempted cardioversion. No patient treated with this strategy had suffered an ischemic stroke at follow-up. Given the substantial increase in demand for an alternative to TEE exclusion of cardiac thrombi in recent years, robust confirmation of the present observation, suggesting CT as a viable option in the ED, is required.

## Figures and Tables

**Table 1:** Characteristics of the study population. Data are presented as median with IQR or absolute number (n) with relative frequency (%). Abbreviations: chd: coronary heart disease; copd: chronic obstructive pulmonary disease.

Baseline Characteristics	LAAT (n = 8)	No LAAT (n = 226)	Overall (n = 234)
male, n %	5 (63)	138 (61)	143 (61)
age, median (IQR)	72 (64–75)	67 (57–76)	68 (57–76)
bmi, median (IQR)	29 (29–32)	28 (24–31)	28 (24–31)
Vitals			
heart rate, median (IQR)	132 (122–167)	140 (123–160)	140 (122–160)
blood pressure, systolic, median (IQR)	164 (162–180)	129 (116–142)	130 (116–147)
blood pressure, diastolic, median (IQR)	113 (70–120)	82 (73–95)	83 (72–96)
Medical history			
heart failure, n (%)	1 (13)	29 (13)	30 (13)
arterial hypertension, n (%)	3 (38)	126 (56)	129 (55)
chd, n (%)	2 (25)	41 (18)	43 (18)
myocardial infarction, n (%)	1 (13)	15 (7)	16 (7)
stroke, n (%)	2 (25)	5 (2)	7 (3)
cerebral vascular disease, n (%)	1 (13)	8 (4)	9 (4)
diabetes mellitus, n (%)	1 (13)	27 (12)	28 (12)
copd, n (%)	1 (13)	19 (8)	20 (9)
hyperlipidemia, n (%)	3 (38)	65 (29)	68 (29)

**Table 2:** AF characteristics. Data are presented as median with IQR or absolute number (n) with relative frequency (%). Abbreviations: oac: oral anticoagulation; CHA_2_DS_2_-VASc: congestive heart failure, hypertension, age ≥ 75 years, diabetes mellitus, stroke, vascular disease, age 65–74 years, sex category.

AF Characteristics	LAAT (n = 8)	No LAAT (n = 226)	Overall (n = 234)
onset unknown, n (%)	6 (75)	135 (60)	141 (60)
first episode, n (%)	3 (38)	105 (46)	108 (46)
CHA_2_DS_2_-VASc, median (IQR)	3 (3–4)	2 (1–4)	2 (1–4)
Oac and/or antiplatelet therapy at admission, n (%)	3 (38)	87 (38)	90 (38)
sinus rhythm restored, n (%)	1 (13)	163 (72)	164 (70)

**Table 3:** Outcomes. Data are presented as median with IQR or absolute number (n) with relative frequency (%).

Outcomes and Follow Up	LAAT (n = 8)	No LAAT (n = 226)	Overall (n = 234)
stroke rate at follow up, n (%)	0 (0)	0 (0)	0 (0)
death, n (%)	0 (0)	14 (6)	14 (6)
follow up completed, n (%)	8 (100)	208 (92)	216 (92)
follow up time, median (IQR)	376 (315–1029)	523 (158–1396)	506 (159–1391)

## Data Availability

The data underlying this article will be shared on reasonable request to the corresponding author.
